# Modelling of inquiry diagnosis for coronary heart disease in traditional Chinese medicine by using multi-label learning

**DOI:** 10.1186/1472-6882-10-37

**Published:** 2010-07-20

**Authors:** Guo-Ping Liu, Guo-Zheng Li, Ya-Lei Wang, Yi-Qin Wang

**Affiliations:** 1 Laboratory of Information Access and Synthesis of TCM Four Diagnosis, Basic Medical College, Shanghai University of Traditional Chinese Medicine, Shanghai 201203, China; 2 The Key Laboratory of Embedded System and Service Computing, Ministry of Education, Department of Control Science & Engineering, Tongji University, Shanghai 201804, China

## Abstract

**Background:**

Coronary heart disease (CHD) is a common cardiovascular disease that is extremely harmful to humans. In Traditional Chinese Medicine (TCM), the diagnosis and treatment of CHD have a long history and ample experience. However, the non-standard inquiry information influences the diagnosis and treatment in TCM to a certain extent. In this paper, we study the standardization of inquiry information in the diagnosis of CHD and design a diagnostic model to provide methodological reference for the construction of quantization diagnosis for syndromes of CHD. In the diagnosis of CHD in TCM, there could be several patterns of syndromes for one patient, while the conventional single label data mining techniques could only build one model at a time. Here a novel multi-label learning (MLL) technique is explored to solve this problem.

**Methods:**

Standardization scale on inquiry diagnosis for CHD in TCM is designed, and the inquiry diagnostic model is constructed based on collected data by the MLL techniques. In this study, one popular MLL algorithm, ML-kNN, is compared with other two MLL algorithms RankSVM and BPMLL as well as one commonly used single learning algorithm, k-nearest neighbour (kNN) algorithm. Furthermore the influence of symptom selection to the diagnostic model is investigated. After the symptoms are removed by their frequency from low to high; the diagnostic models are constructed on the remained symptom subsets.

**Results:**

A total of 555 cases are collected for the modelling of inquiry diagnosis of CHD. The patients are diagnosed clinically by fusing inspection, pulse feeling, palpation and the standardized inquiry information. Models of six syndromes are constructed by ML-kNN, RankSVM, BPMLL and kNN, whose mean results of accuracy of diagnosis reach 77%, 71%, 75% and 74% respectively. After removing symptoms of low frequencies, the mean accuracy results of modelling by ML-kNN, RankSVM, BPMLL and kNN reach 78%, 73%, 75% and 76% when 52 symptoms are remained.

**Conclusions:**

The novel MLL techniques facilitate building standardized inquiry models in CHD diagnosis and show a practical approach to solve the problem of labelling multi-syndromes simultaneously.

## Background

Coronary heart disease (CHD) is a common cardiovascular disease that is extremely harmful to humans. It is easier to find in middle and old aged people with high mortality. CHD belongs to the scope of chest heartache in Traditional Chinese Medicine (TCM); there have been extensive experiences in the diagnosis and treatment of CHD in TCM and the therapeutic effects are fairly satisfying [1]. However, TCM describes diseases by qualitative and fuzzy quantitative words: there is no clear functional relationship between the symptoms and syndromes. Currently, searching the objective and inherent relationship between the symptoms and syndromes, followed by constructing diagnostic models of syndromes is a fast developing field. Standardization and objectification of TCM diagnosis is an important and urgent task, which could also be fatal in scientific research, teaching and clinical practice [2].

However, there are few systematic studies of quantitative diagnosis for CHD, especially of the standardization study of inquiry diagnosis for CHD. For example, Jia et al. had investigated the contribution of symptoms to syndromes diagnosis by using a complex system focused on entropy [3]. Many authors had worked on the diagnostic standardization of many other diseases, and various techniques of multivariate statistics have been applied in the construction of diagnostic models in TCM, such as discriminant analysis and regression analysis in the diagnosis of blood stasis syndrome [4,5] and stroke [6,7]. Although multivariate statistics has some superiority in the solution of quantitative diagnosis in TCM, the problem on clinical data analysis with high nonlinearity could not be solved by these techniques. Moreover, the complex interaction among different symptoms could not be reflected clearly, and the diagnostic rules of TCM could not be revealed comprehensively and widely. In this circumstance, non-linear data mining techniques are appealable in quantitative diagnosis, [8].

With the introduction of data mining techniques, investigators have applied several non-linear learning techniques into the research of diagnostic standardization and objectification in TCM, such as k nearest neighbour (kNN), neural networks, Bayesian networks, structure equations, decision tree, genetic algorithm, etc. Some authors introduced the structure equations model into the syndrome study of chronic atrophic gastritis in TCM, and they demonstrated that the most common symptoms of the disease in TCM are related with corresponding diagnostic indicators in accordance with clinical practice in TCM [9]. Other authors introduced Bayesian networks into the clinical analysis of blood stasis syndrome and the quantitative diagnosis with results of high accuracy [10]. An improved conjugate gradient learning algorithm was introduced into the construction of three layer forward BP network model in diabetic nephropathy with satisfied forecast results [11]. The diagnostic criterion for deficiency of yin syndrome and endothelial dysfunction symptoms by the complex system technique based on entropy was investigated, and the result demonstrated that coincidence rate was good [12].

In the above mentioned syndrome standardization and objectification studies, most of the algorithms are to solve problems of single syndrome diagnosis, i.e., single label learning. However, in clinical practice, many symptoms are presenting various syndromes. Previous studies [13] have shown that the main syndromes of CHD are deficiency accompanying with excess, e.g. deficiency of qi syndrome and blood stasis syndrome, deficiency of qi syndrome and turbid phlegm syndrome, deficiency of yang syndrome and turbid phlegm syndrome, blood stasis syndrome and Qi stagnation syndrome, as the predominant combining forms of their syndromes. But the aforementioned data mining algorithms could not forecast so many syndromes, i.e., multiple labels simultaneously. Compared with conventional learning methods, multi-label learning could identify syndrome information in TCM more effectively, and could solve the multi-label problems of one sample with several syndromes. In clinical syndrome diagnostic, the inquiry information occupies by 80%. Thus the MLL technique is investigated in modelling of syndrome diagnosis of CHD and doing forecast in syndrome inquiry diagnosis.

In this paper, standardization scale of inquiry information is designed; 555 cases of CHD are collected following this scale, and the clinical diagnosis is performed. Based on the data set, models for multi syndromes are constructed by multi-label learning, and the influence of symptom selection on modelling is also assessed. In the Method Section, we introduce the data collecting method, kNN and the multi-label learning ML-kNN algorithms; in the Results Section, the results of models constructed based on kNN and ML-kNN is studied and the influence of symptom selection on models is assessed. In the Discussion Section, the reason why multi-label learning could improve the results is clarified. Eventually, we summarize the paper in the Conclusion Section.

## Methods

### Data set of coronary heart disease in TCM

In this paper, a heart system inquiry diagnosis scale [14] is designed, in which the symptoms are defined clearly, and the detailed collecting methods are listed. The scale is shown in [Additional file 1].

Inclusion criteria of the patients are: 1) The patients who meet the diagnostic criteria of CHD; 2) The patients who are informed consented.

Diagnosis criteria of the patients are in western medicine and TCM. Diagnosis criteria in western medicine are by referring to "Naming and diagnosis criteria of ischemic heart disease" issued by International Society of Cardiology and the Joint Subject Team on standardization of clinical naming in World Health Organization [15]. CHD is defined as a stenosis greater than 50% in at least one of 16 segments of the 3 major coronary arteries and their branches by coronary angiography. Diagnosis criteria in TCM are according to the "Differentiation standards for symptoms and signs of Coronary Heart Disease and Angina Pectoris in Traditional Chinese Medicine" in the "Standards for differentiation of chest pain, chest distress, palpitation, short breath or debilitation for coronary heart disease in Traditional Chinese Medicine" modified by China Society of Integrated Traditional Chinese and Western Medicine in 1990 and the "Guideline for Clinical study of new drugs in Chinese herbs", and the standards in textbooks [16,17]. After discussion with experts in cardiology, the diagnosis criteria are established.

Exclusion criteria are 1) The patients with mental diseases or with other severe diseases; 2) The patients who could not express their feeling clearly; 3) The patients who refused to participate in our study or without informed consent.

The patients with coronary heart disease are selected in Cardiology Department of Longhua Hospital Affiliated to Shanghai University of Traditional Chinese Medicine, Shuguang Hospital Affiliated to Shanghai University of Traditional Chinese Medicine, Shanghai Renji Hospital and Shanghai Hospital of Chinese Medicine. The cases with incomplete information or inconformity with the diagnosis criteria of CHD are removed. This work has been approved by the Shanghai society of medical ethics. All the patients have signed the informed consent form. Finally, a total of 555 cases are obtained in the study.

Three senior chief TCM physicians performed diagnosis individually for the 555 cases by referring to the diagnosis criteria established in the study, and the data with consistent results between 2 physicians are recorded; as for the inconsistent results, the data is not recorded until the result is consistent after discussion with the other experts.

Among the 555 patients, 265 patients are male (47.7%, with mean age of 65.15 +/- 13.17), and 290 patients are female (52.3%, with mean age of 65.24 +/- 13.82). The symptoms collected for inquiry diagnosis include 8 dimensions: cold or warm, sweating, head, body, chest and abdomen, urine and stool, appetite, sleeping, mood, and gynaecology, a total of 125 symptoms. There are 15 syndromes in differentiation diagnosis, of which 6 commonly-used patterns are selected in our study, including: z1 Deficiency of heart qi syndrome; z2 Deficiency of heart yang syndrome; z3 Deficiency of heart yin syndrome; z4 Qi stagnation syndrome; z5 Turbid phlegm syndrome and z6 Blood stasis syndrome.

The data set is shown in [Additional file 2].

### Computational methods

In our study, we construct models of the relationship between symptoms and syndromes of inquiry diagnosis by means of the multi-label k-nearest neighbour (ML-kNN) algorithm. Then the results are compared with those calculated by the classical k-nearest neighbour (kNN) algorithm.

kNN is an algorithm whose idea is to search for the nearest point in training data set [18]. This theory regards an instance as a point in synthesis space; thus, the label of a test instance is probably similar to those of several nearest points. Based on this theory, the algorithm of kNN is to search for k train instances nearest to the test instance, then according to their labels, to predict the label of the test instance. Compared with other mining algorithms, the advantage of kNN lies in simpler training process, better efficiency and forecast accuracy.

In this paper, the data set for differentiation diagnosis of CHD belongs to multi-label; whereas kNN only processes single label data sets, so the collected data set should be split into many groups of single label to be calculated. The modified algorithm is shown as below.

Step 1: Split the data set with n labels into n data sets with single label.

Step 2: Presuming there are p samples in the test instance, for each instance x_i_, calculate the distances of x_i _to all training instances; for each instance x_i _of test data, find the k instances of training data with the smallest distance.

Step 3: According to the labels of k instances, forecast the label of each test instance x_i _(in this paper, the result is judged as positive if the number of labels is more than k/2, otherwise, negative). Then the forecast results of p test instances are obtained.

Step 4: Combine n groups of results to obtain the forecast result of multiple labels and assess the forecast results according to multiple label evaluation criteria.

In clinical practice, there are many multi-label problems similar to the modelling for symptoms of inquiry diagnosis in our paper. If a single label algorithm is used, the multiple labels are usually divided and then calculated. In the multi-label data, there is much relationship among each label, so simple splitting inevitably result in data loss. For this reason, multi-label learning algorithms are developed so as to better reveal the correlation of the labels, of which multi-label kNN (ML-kNN) is a popular technique [19,20]. ML-kNN is a lazy multi-label learning algorithm developed on the basis of kNN. Based on the theory of kNN, ML-kNN aims to find k nearest instances for each test instance. In ML-kNN, the labels of test instances are judged directly by nearest instances, which is different from kNN. The algorithm is shown as below.

Step 1: Calculate the conditional probability distribution of each instance associated to each label;

Step 2: Calculate the distance between the x_i _test instance and the training instances; then find k nearest instances for x_i_. Repeat for each test instance.

Step 3: According to the labels of k training instances and the conditional probability associated to each label, forecast the probability of the x_i _instance and then acquire the forecast results (here ≥ 0.5 is taken); Repeat for each test instance.

Step 4: Evaluate the forecast results according to multi label evaluation criteria.

Codes of both algorithms of ML-kNN and kNN are implemented on the MATLAB platform, which are shown in [Additional file 3].

To make the computational results more fruitful, the results obtained by ML-kNN and kNN are compared with those of two other multi-label learning algorithms, RankSVM [21] and BPMLL [22]. We used the default and experienced parameters values in RankSVM and BPMLL. For RankSVM, the number of hidden neurons is 8, the max training epochs is 6. For BPMLL, the number of max iteration is 10. Parameters not mentioned were set to the default values [21,22].

### Experimental design and evaluation

In the CHD data set, 90% of the samples are randomized as the training set and the other 10% are as the test set. The forecast analysis of models for syndromes of inquiry diagnosis in TCM is performed after re-testing the models for 50 times and taking the mean value. Then different values of k are chosen to evaluate its influence on kNN and ML-kNN. In this paper, k is chosen from {1, 3, 5, 7, 9, 11}. According to the frequency of symptoms, the symptom features are removed as follows: the symptoms with frequencies of {≤ 10, ≤ 20, ≤ 40, ≤ 70, ≤ 100, ≤ 150, ≤ 200 and ≤ 400} are removed in turn, thus the symptom subsets with 150, 106, 83, 64, 52, 32 and 21 are obtained. Models of inquiry diagnosis are constructed on the basis of symptom subsets, and the influence of symptom selection on forecast model of inquiry diagnosis is investigated.

Let *X *denote the domain of instances and let *Y *= {1,2,⋯,Q} be the finite set of labels. Given a training set T = {(x_1_, Y_1_), (x_2_, Y_2_), ⋯, (x_m_, Y_m_)}(x_i _∈ *X*, Y_i _⊆ *Y*), the goal of the learning system is to output a multi-label classifier h: *X*→2^*y *^which optimizes some specific evaluation metric. It is suppose that, given an instance x_i _and its associated label set Y_i_, a successful learning system will tend to output larger values for labels in Y_i _than those not in Y_i_, i.e. f(x_i_, y_1_) >*f*(x_i_, y_2_) for any y_1 _∈ Y_i _and y_2 _∉ Y_i_.

The real-valued function f(·,·) can be transformed to a ranking function rank_f_(·,·), which maps the output of f(x_i_, y) for any y ∈ *Y *to {1,2,⋯,Q} such that if f(x_i_, y_1_) >*f*(x_i_, y_2_) then rank_f_(x_i_, y_1_) < rank_f_(x_i_, y_2_).

To evaluate the forecast results of ML-kNN comparing with traditional kNN, the following criteria [19] are used:

• **Average_Precision **evaluates the average fraction of syndrome labels ranked above a particular label which actually is in the label set as follows:

The performance is perfect when it is 1; the bigger the value of average precision, the better the performance of classifiers.

• **Coverage **evaluates how far we need, on the average, to go down the list of syndrome labels in order to cover all the proper labels of the instance as follows:

It is loosely related to precision at the level of perfect recall. The smaller the value of coverage, the better the performance.

• **Ranking_Loss **evaluates the average fraction of syndrome label pairs that are reversely ordered for the instance as follows:

where z means  denotes the complementary set of Y in *Y*.

The performance is perfect when it is 0; the smaller the value of ranking loss, the better the performance.

## Results

### Forecast results of syndrome models for inquiry diagnosis

**Figure 1:**
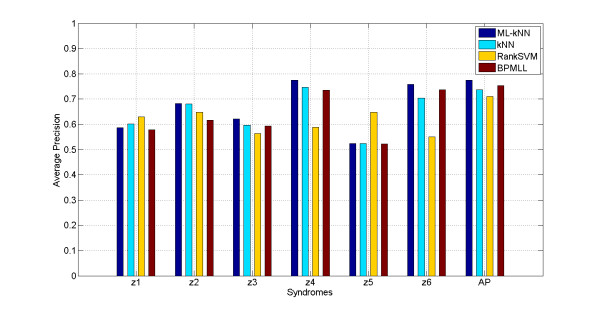
Results of average_precision obtained in syndrome models by using ML-kNN, RankSVM, BPMLL and kNN with k = 5

On the data set with all symptoms, taken k = 5, the models of ML-kNN are built as described in the Methods Section. The mean accuracy obtained on the 6 syndrome labels is shown in Figure 1, and the results of kNN, RankSVM and BPMLL are also shown in the Figure as a comparison, where the horizontal coordinate stands for the labels of syndromes forecasted and AP means the average results of the whole labels; the longitudinal coordinate stands for forecast accuracy with 100% as the highest value. The comparative results of ML-kNN, RankSVM, BPMLL and kNN with k = 5 under three evaluation criteria are listed in Table 1.

**Table 1:** Results of syndrome models for inquiry diagnosis by using ML-kNN, RankSVM, BPMLL and kNN with k = 5

Evaluation criteria	ML-kNN	kNN	RankSVM	BPMLL
Average_Precision(%)	77.4 ± 3.3	73.6 ± 3.1	71.0 ± 2.1	75.4 ± 2.7

Coverage	3.31 ± 0.31	3.44 ± 0.30	3.69 ± 0.28	3.36 ± 0.33

Ranking_Loss	0.283 ± 0.035	0.386 ± 0.037	0.419 ± 0.041	0.311 ± 0.039

The results in Figure 1 and Table 1 demonstrate that: 1) Comparing the forecast results of syndrome models by using ML-kNN with those by kNN, RankSVM and BPMLL on the whole, the Average_Precision result of ML-kNN is 3.8%, 6.4% and 2.0% more than that of kNN, RankSVM and BPMLL, while the Coverage result of ML-kNN are 0.13, 0.38 and 0.05 lower than kNN, RankSVM and BPMLL, the Ranking_Loss result of ML-kNN are 0.103, 0.136 and 0.028 lower than kNN, RankSVM and BPMLL, respectively. According to the aforementioned evaluation criteria, the higher of Average_Precision, the better results obtained, and other measures are just the opposite. Thus the ML-kNN results are significantly better than kNN, RankSVM and BPMLL. 2) As multi-label algorithms, BPMLL obtains better results than kNN, while RankSVM does not. 3) On each label, five out of six syndromes of ML-kNN have better forecast accuracy; the accuracy of ML-kNN is some lower than kNN and RankSVM only in z1 and z4 syndrome. 4) Different labels result in different forecast results; although clinical values are shown from the results on the whole, the result of z5 is slightly greater than 50%.

### Influence on the forecast results by using different k values

**Figure 2:**
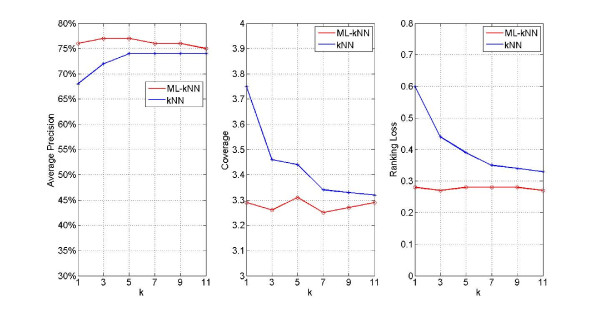
Results of syndrome models for inquiry diagnosis on whole labels by using ML-kNN and kNN with different k values

In order to determine whether the k value influenced the forecast results in ML-kNN, we construct models with k values as 1, 3, 5, 7, 9 and 11, respectively. Then the forecast results are listed in Figure 2 under the evaluation criteria of Average_Precision, Coverage and Ranking_Loss, respectively, where the horizontal coordinate stands for the k value, and longitudinal coordinate stands for results of Average_Precision, Coverage and Ranking_Loss, respectively.

From Figure 2, it can be seen that: 1) the forecast results of ML-kNN and kNN vary with variation of k values, but the change is small with a minor impact on diagnostic results with higher k values, suggesting that both algorithms are stable. 2) Whatever k values, the forecast Average_Precision results of ML-kNN are significantly higher than that of kNN, while the results of Coverage and Ranking_Loss are significantly lower than kNN, suggesting that the modelling results of ML-kNN are better than those of kNN. 3) On the criterion of Average_Precision, when taking k value as 5, both algorithms obtain the best forecast results. This propensity varies on the other two criteria. On the Coverage criterion, the best results are obtained when taking k as 7 and 9, in ML-kNN and kNN, respectively. While on Ranking_Loss, the optimal results are obtained taking k as 3 and 11, respectively.

**Figure 3:**
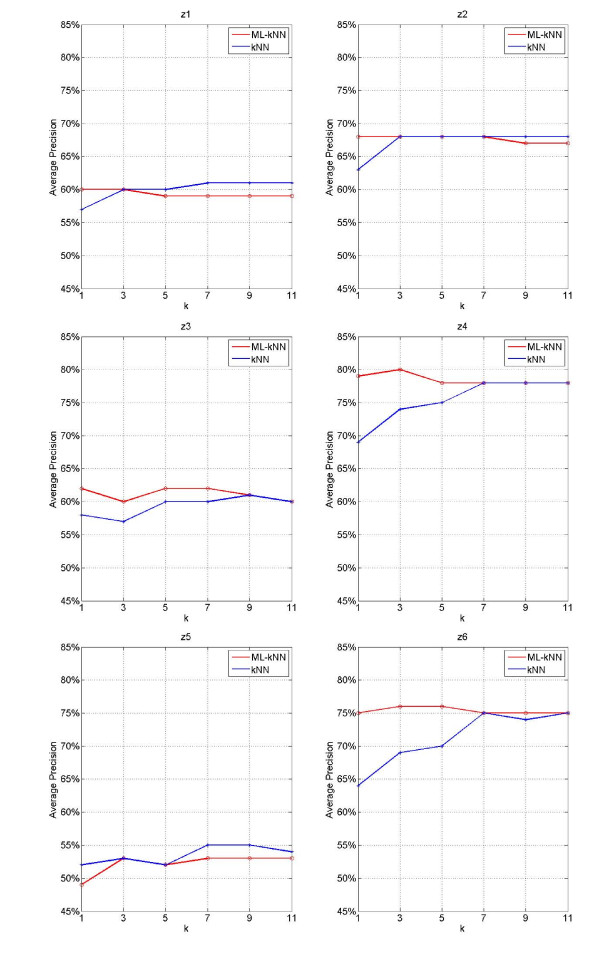
Results of syndrome models for inquiry diagnosis on each label by using ML-kNN and kNN with different k values

In order to further investigate the situations of each label, the forecast results of Average_Precision on different syndrome labels by using ML-kNN and kNN with different k values are illustrated in Figure 3, where the horizontal coordinate stands for the k value, and longitudinal coordinate stands for results of Average_Precision.

From Figure 3, we may find: 1) Forecast Average_Precision of ML-kNN reaches the highest mostly when k = 3; and with the increase of k value, the forecast accuracy decreases; 2) Forecast results of Average_Precision by using kNN increases with k value increasing, with the highest accuracy when k = 7; then it maintains steady with k value increasing; 3) As for the forecast of syndromes, some are better in ML-kNN and some are better in kNN; on the whole, ML-kNN is better than kNN.

### Influence of symptom selection on the forecast results

In our study, the symptoms in inquiry diagnosis are comprehensive, with a total of 125 symptoms. The frequency of some symptoms is high and others are low. Some symptoms influence the diagnosis of CHD to a greater extent and some to a less extent. Consequently, we select symptoms according to the frequency of symptoms, and choose the symptoms with higher frequency to investigate the influence of symptom selection on modelling.

According to the frequency of symptoms, we choose 125 symptom subset (removing those with frequency ≤ 10), 106 symptom subset (removing those with frequency ≤ 20), 83 symptom subset (removing those with frequency ≤ 40), 64 symptom subset (removing those with frequency ≤ 70), 52 symptom subset (removing those with frequency ≤ 100), 32 symptom subset (removing those with frequency ≤ 150), and 21 symptom subset (removing those with frequency ≤ 200 and ≤ 400). Finally, 7 subsets of symptom are obtained. On the 7 subsets, respective models are constructed by using ML-kNN, kNN, RankSVM and BPMLL, whose forecast results of three criteria are listed in Table 2.

**Table 2:** Results of syndrome models for inquiry diagnosis on total labels by using ML-kNN, RankSVM, BPMLL and kNN with different symptom subsets

symptoms	Average_Precision(%)			
	
	ML-kNN	kNN	RankSVM	BPMLL
125	76.2 ± 3.1	74.2 ± 3.3	70.9 ± 3.1	76.1 ± 3.8

106	76.6 ± 2.7	73.7 ± 3.3	71.0 ± 3.4	75.0 ± 3.3

83	76.8 ± 2.4	75.0 ± 3.1	74.3 ± 2.9	75.8 ± 3.4

64	76.6 ± 2.9	75.3 ± 2.9	74.4 ± 2.8	73.9 ± 3.9

52	78.0 ± 2.4	74.7 ± 2.3	73.3 ± 2.6	75.1 ± 2.7

32	75.7 ± 3.2	73.7 ± 3.5	72.1 ± 2.9	75.0 ± 2.7

21	74.9 ± 2.9	73.2 ± 3.8	70.5 ± 3.5	74.4 ± 3.3

symptoms	Coverage			
	
	ML-kNN	kNN	RankSVM	BPMLL

125	3.28 ± 0.32	3.44 ± 0.23	3.47 ± 0.28	3.30 ± 0.35

106	3.28 ± 0.27	3.41 ± 0.31	3.43 ± 0.28	3.52 ± 0.32

83	3.29 ± 0.28	3.46 ± 0.28	3.38 ± 0.29	3.32 ± 0.38

64	3.22 ± 0.23	3.43 ± 0.23	3.48 ± 0.29	3.41 ± 0.28

52	3.21 ± 0.24	3.43 ± 0.21	3.38 ± 0.35	3.34 ± 0.27

32	3.25 ± 0.31	3.49 ± 0.35	3.41 ± 0.25	3.43 ± 0.23

21	3.26 ± 0.32	3.51 ± 0.35	3.53 ± 0.36	3.42 ± 0.35

symptoms	Ranking_Loss			
	
	ML-kNN	kNN	RankSVM	BPMLL

125	0.290 ± 0.031	0.394 ± 0.044	0.384 ± 0.032	0.291 ± 0.036

106	0.283 ± 0.029	0.390 ± 0.037	0.351 ± 0.035	0.311 ± 0.031

83	0.277 ± 0.024	0.388 ± 0.037	0.329 ± 0.031	0.337 ± 0.029

64	0.266 ± 0.032	0.384 ± 0.042	0.348 ± 0.040	0.330 ± 0.027

52	0.271 ± 0.028	0.379 ± 0.034	0.353 ± 0.036	0.309 ± 0.048

32	0.273 ± 0.047	0.402 ± 0.036	0.343 ± 0.042	0.294 ± 0.029

21	0.279 ± 0.041	0.414 ± 0.029	0.369 ± 0.044	0.321 ± 0.037

Table 2 demonstrates that different symptom subsets leads to different forecast accuracy, but whatever the subset is, ML-kNN is superior to RankSVM, BPMLL and kNN. By using both algorithms, the forecast Average_Precision on the subset of 52 symptoms is the highest: 78.0% by ML-kNN, 74.7% by kNN, 73.3% by RankSVM, and 75.1% by BPMLL. As to other two evaluation measures Coverage and Ranking_Loss, ML-kNN is also superior to RankSVM, BPMLL and kNN.

**Figure 4:**
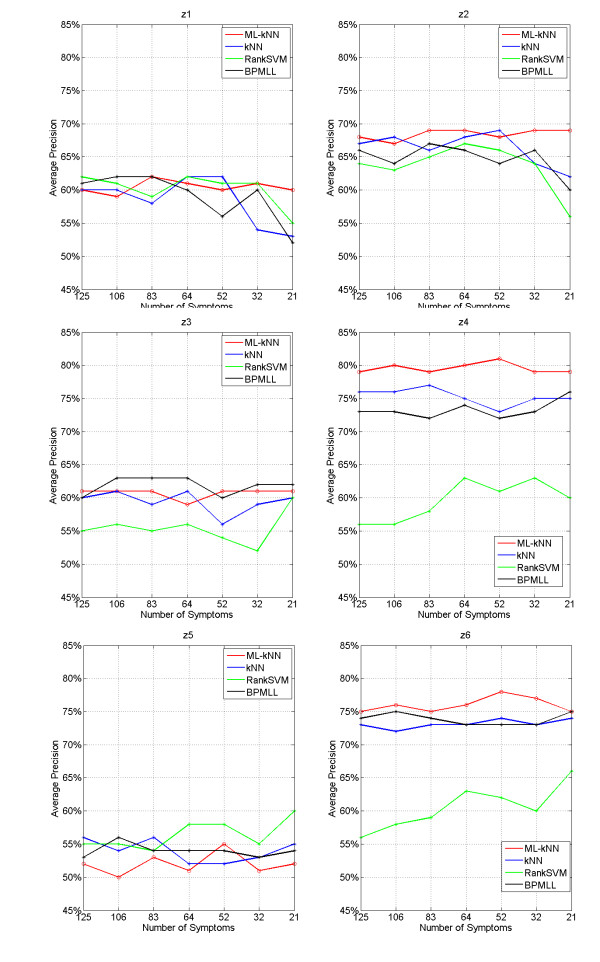
Results of syndrome models for inquiry diagnosis on each label by using ML-kNN, kNN, RankSVM and BPMLL with different symptom subsets

Moreover, the forecast results for each syndrome are illuminated in Figure 4, where the horizontal coordinate stands for symptom subsets, and longitudinal coordinate stands for forecast accuracy. In order to compare ML-kNN, RankSVM, BPMLL and kNN accurately, the detailed results of ML-kNN, RankSVM, BPMLL and kNN on the optimal subset of 52 symptoms are listed in table 3.

**Table 3:** Results of syndrome models for inquiry diagnosis on each label by using ML-kNN, RankSVM, BPMLL and kNN on the 52-symptom subset

Syndromes	ML-kNN(%)	kNN(%)	RankSVM(%)	BPMLL(%)
z1	60.3 ± 2.7	61.8 ± 2.4	61.1 ± 2.9	55.5 ± 3.4

z2	67.8 ± 3.1	68.8 ± 3.6	65.7 ± 3.5	63.5 ± 3.4

z3	61.1 ± 3.3	55.5 ± 3.5	54.4 ± 3.8	60.3 ± 3.7

z4	81.2 ± 2.4	73.4 ± 3.2	61.2 ± 4.6	71.6 ± 2.5

z5	54.9 ± 1.8	52.3 ± 2.3	58.0 ± 2.4	53.1 ± 2.9

z6	78.4 ± 4.1	73.9 ± 4.7	61.8 ± 4.9	72.7 ± 4.3

Figure 4 shows that different symptom subsets result in different forecast accuracy; ML-kNN is rather steady without much fluctuation. Table 3 demonstrates that out of the six syndromes, four syndromes z3 Deficiency of heart yin syndrome, z4 Qi stagnation syndrome, z5 Turbid phlegm syndrome and z6 Blood stasis syndrome are better by ML-kNN than by kNN; while their forecast accuracy in the other two syndromes z1 Deficiency of heart qi syndrome and z2 Deficiency of heart yang syndrome is slighter lower by ML-kNN than by kNN. The forecast accuracy in the z5 Turbid phlegm syndrome is lower by ML-kNN than by RankSVM and BPMLL. As a whole, the forecast results of ML-kNN are superior to RankSVM, BPMLL and kNN.

## Discussions

### Modelling by using multi-label learning

K nearest neighbour (kNN) is widely used in biomedical data mining field. As an extreme of minimal distance classifiers, kNN is simple, easy and efficient. However, when determining the label of a test sample, kNN can only do forecast once at a time, i.e., classify a syndrome to one category in TCM. But in clinical practice, there may be strong relevance among different syndromes. The syndrome of one patient mainly is the composition of several syndromes, e.g. deficiency of heart qi syndrome usually exists with qi stagnation syndrome, turbid phlegm syndrome and/or blood stasis syndrome. Due to the defects of kNN and other conventional single label mining techniques, we recommend applying novel multi-label mining algorithm ML-kNN.

Classification forecast is performed on 555 cases of inquiry diagnosis for CHD in TCM, with four algorithms, multi-label ML-kNN, RankSVM and BPMLL as well as single label kNN. Both algorithms of ML-kNN and kNN are compared by using different k values and different symptom subsets. When k = 5, the Average_Precision of ML-kNN is 77.4%, significantly superior to 73.6% of kNN, 71.0% of RankSVM and 75.4% of BPMLL. On the optimal 52 symptom subset, the Average_Precision of ML-kNN is 78.0%, significantly superior to 74.4% of kNN, 73.3% of RankSVM and 75.1% of BPMLL too. Similarly, the forecast accuracy of ML-kNN is obviously better than kNN, RankSVM and BPMLL.

In the kNN algorithm, the syndrome diagnosis of CHD is split into several single label problems to perform forecast, then the results are combined. In this process, the latent relationships among labels (syndromes) are lost. Nevertheless, in the ML-kNN algorithm, several labels (syndromes) are treated as a whole, and the relationships among labels (syndromes) are retained. Consequently, the forecast accuracy of ML-kNN is theoretically better than that of kNN, which is also confirmed by our experiment. Meanwhile, the comparison between ML-kNN and kNN reveals the mutual relationship between symptoms in TCM. The results suggest that when performing classification forecast for data sets of inquiry diagnosis for CHD, ML-kNN achieves better results and it seems to be a practicable solution for multi-label problems in clinical diagnosis in TCM at present.

### The Optimal symptom subset

The aim of symptom feature selection is to reduce dimension of the symptoms in inquiry diagnosis for CHD, and to find the most related symptom subsets. Our results demonstrate that the forecast accuracy is improved after symptom selection either by ML-kNN, kNN, RankSVM or BPMLL. Furthermore, an optimized 52-symptom subset is obtained after symptom selection, shown in Table 4.

**Table 4:** Frequency distribution of the optimal 52-symptom subset

No.	Symptoms	Frequency	No.	Symptoms	Frequency
1	X6 Duration of pain seizure	454	27	X23 Tinnitus	195

2	X8 Relieving factor	442	28	X15 Fear of cold	194

3	X2 Chest oppression	436	29	X28 Cough	181

4	X5 Seizure frequency	424	30	Y52 Frequent seizure	181

5	X4 Short breath/dyspnea/suffocation	387	31	X75 Impetuosity and susceptibility to rage	179

6	X7 Inducing (aggravating) factor	380	32	X72 The frequent and increased urination at night	164

7	X10 Hypodynamia	363	33	Y317 Fixed pain	146

8	X1 Palpitation	358	34	X48 Thirst with preference for hot water	144

9	Y31 Pain location	348	35	Y73 Aggravating gloom	142

10	X40 Soreness and weakness of waist and knees	282	36	X29 Cough with sputum	141

11	X3 Chest pain	270	37	X13 Amnesia	135

12	X44 Thirsty and dry pharynx	270	38	X9 Edema	134

13	X22 Dizziness and Blurred vision	269	39	X311 Xuli - the apex of the heart	131

14	Y82 Relieving after administration of drug	260	40	Y731 The condition of difficult in falling asleep	130

15	Y61 Transient	257	41	X62 Constipation	126

16	Y72 Inducing (aggravating) after movement	251	42	X291 Color of sputum	124

17	Y51Occasional seizure	245	43	X16 Cold limbs	123

18	Y81 Relieving after rest	242	44	X292 Character of sputum	120

19	X73 Insomnia	241	45	X49 Poor appetite and less amount of food	118

20	X11 Dysphoria	224	46	X32 Gastric stuffiness	105

21	X79 Menopause	222	47	X45 Absence of thirst and no desire for water drink	105

22	X20 Spontaneous sweating	217	48	Y75 Inducing (aggravating) when cloudy or rainy	103

23	X41 Numbness of hands and feet	206	49	X53 Bitter taste	102

24	Y32 Character of pain	205	50	Y294 Difficulty or easy level of coughing with sputum	101

25	X21 Night sweat	201	51	Y71 Seizure when quiet or without inducing factor at night	101

26	Y62 Persistent seizure	198	52	X27 Sore-throat	100

CHD belongs to the scope of heart diseases family in TCM. The main physiological functions of the heart are to control the blood vessels and govern the mind. In TCM, heart diseases result in dysfunction of blood vessels and bring on symptoms such as chest pain, choking sensation in chest, palpitation and numb of hands or feet. Dysfunction of "governing the mind" results in symptoms such as insomnia, anxiety and amnesia. In the TCM, heart is the monarch organ: it pumps blood to warm the body, and thereby it pertains to fire. Therefore, heart diseases may lead to decreased function of warm, presenting chilly or cold limb, soreness and weakness of waist and knees and night-time frequency or nocturia. Heart dominates sweat in secretion, so heart diseases may lead to self-sweating and night sweat. If the heart-fire flames up with liver-fire, it may cause irritability, impatience and bitter taste. Heart and spleen are the mother-child relationship, so heart disease may result in spleen disease and bring on the symptoms like anorexia, eating less, abdominal fullness and distension.

The duration, inducing (or aggravating) factors and relieving factors of chest pain are the main factors to determine the feature of CHD. As shown in Table 4, the above-mentioned symptoms are the main information for diagnosis of CHD. Basically, most of the information about general pathology of CHD is listed in the table, and these items comprise the optimal symptom subset. Our results suggest that combination of symptom feature selection with classification algorithms could simplify symptom information and further improve both the comprehension and forecast accuracy of the syndromes of CHD.

Symptom feature selection by using frequency should be improved. Two cases take place out of expectation. One case is that symptoms are frequent, but they are meaningless, such as menopause. Since the average age of the females in this work is higher than 65, so most of these females are menopause, but this has little relation with CHD. The other case is that symptoms are rare, but it is important information to diagnose the CHD, such as migratory pain. It is critical to determine the syndrome of Qi stagnation, which is removed for its low frequency. We hope to enlarge the samples and continue the study on other symptom selection methods.

## Conclusions

A multi-label learning algorithm ML-kNN is employed to construct the syndrome models of inquiry diagnosis for CHD in TCM, and further produces better results than RankSVM, BPMLL and kNN do by means of three criteria of Average_Precision, Coverage and Ranking_Loss. ML-kNN not only classifies the syndromes of inquiry diagnosis for CHD, but also solves the multi-label problems of one sample with several syndromes simultaneously. It overcomes the defect of conventional single label mining algorithms like kNN and turns out to be an effective technique for solving problems with multiple labels in clinical practice of TCM. Furthermore, combination of symptom selection with multi-label learning algorithms decreases the dimension of symptoms in inquiry diagnosis of CHD and consequently simplifies the symptom information and increases forecast accuracy. The optimal symptom subset obtained by symptom selection could also be used for guidance in clinical practice.

Future works includes designing more effective symptom selection algorithms, and employing the multi-label learning algorithms on more biomedical data sets.

## Competing interests

The authors declare that they have no competing interests.

## Authors' contributions

All authors read and approved the final manuscript. GPL contributed to the collection, sorting, diagnostics of inquiry information of CHD and the article writing. GZL conceived and revised the paper, designed algorithms and experiments. YLW implemented the algorithms and performed the calculation. YQW contributed to the syndrome diagnosis of CHD patients.

## Authors' information

**Guo-Ping Liu **received her Ph.D. degree from Shanghai University of Traditional TCM in 2008, and now is the assistant professor in the Fundamental Faculty of Medical College, Shanghai University of Traditional Chinese Medicine, majoring in the research of objectification of four diagnostics in traditional Chinese medicine and standardization of syndromes in traditional Chinese medicine.

**Guo-Zheng Li **received his Ph.D. degree from Shanghai JiaoTong University in 2004. He is currently an Associate Professor in the Department of Control Science & Engineering, Tongji University, China. He is serving on the Committees at CCF Artificial Intelligence and Pattern Recognition Society, CAAI Machine Learning Society, International Society of Intelligent Biological Medicine and IEEE Computer Society. His research interests include feature selection, classifier design, and machine learning in bioinformatics, traditional Chinese medicine and other intelligent applications. In the recent years, Li has published 50+ refereed papers in prestigious journals and conferences. He is Editors on board of IJDMB, IJMLC, IJAISC, IJFIPM, IJCBDD, JETWI, IJCIBSB and program chair of IJCBS 2009 and ITCM 2010.

**Ya-Lei Wang **is a M.Sc. degree student in Tongji University, majors in multi-label learning and feature selection.

**Yi-Qin Wang **received her Ph.D. degree from Shanghai University of Traditional Chinese Medicine in 2002, and now is the professor in the Fundamental Faculty of Medical College, Shanghai University of Traditional Chinese Medicine, doctoral degree supervisor, majoring in the research of objectification of four diagnostics in Chinese Medicine and standardization of syndromes in Chinese Medicine.

## Pre-publication history

The pre-publication history for this paper can be accessed here:

http://www.biomedcentral.com/1472-6882/10/37/prepub

## Supplementary Material

Additional file 1 **The inquiry diagnostic scale of coronary heart disease in traditional Chinese medicine**. Please refer to the Subsection of Data set of coronary heart disease in TCM in this paper. Click here for file

Additional file 2 **The collected data set of coronary heart disease in traditional Chinese medicine**. Please refer to the Subsection of Data set of coronary heart disease in TCM in this paper. Click here for file

Additional file 3 **Source codes of ML-kNN and kNN in MATLAB language**. Please refer to readme in the zip file. Click here for file
