# Supplementary material for: Modelling of inquiry diagnosis for coronary heart disease in traditional Chinese medicine by using multi-label learning
Source: BMC Complement Altern Med. 2010 Jul 20;10:37. doi: 10.1186/1472-6882-10-37 (PMC2921356; doi:10.1186/1472-6882-10-37)
Supplement: Additional file 1 — The inquiry diagnostic scale of coronary heart disease in traditional Chinese medicine. Please refer to the Subsection of Data set of coronary heart disease in TCM in this paper. [file 1472-6882-10-37-S1.PDF]

# Diagnostic Inquiry Scale for Heart System in TCM

| **Essential information** | **Full name** | | **Gender** | | **Age** | |
|---|---|---|---|---|---|---|
| | **Occupation** | | | **Marriage** | | |

**Chief complaint:** ______________________________________________

**All the symptoms are made as follows : YES=1 ; NO=0**

1. **Key Inquiry**

**X1** Palpitations

**X2** Chest oppression

**X3** Chest pain

**Y31** Pain location:

**Y311** Xuli-the apex of the heart

**Y312** Danzhong

**Y313** Front part of the chest

**Y314** Radiate to shoulders, back and medial arms

**Y315** Other locations

**Y316** Migratory pain

**Y317** Fixed pain

**Y32** Character of pain:

**Y321** Stabbing pain

**Y322** Dull pain

**Y323** Distending pain

**Y324** Colic pain

**Y325** Others

**X4** Short breath/Dyspnea/Suffocation

**X5** Seizure frequency: **Y51** Occasional seizure **Y52** Frequent seizure

**X6** Duration of seizure: **Y61** Transient seizure **Y62** Persistent seizure

**X7** Inducing (aggravating) factor:

**Y71** Seizure when quiet or without inducing factor at night

**Y72** Inducing (aggravating) after movement

**Y73** Inducing (aggravating) after gloom

**Y74** Inducing (aggravating) after drinking

**Y75** Inducing (aggravating) when cloudy or rainy

**Y76** Inducing (aggravating) when cold suddenly

**X8** Relieving factor:

**Y81** Relieving after rest

**Y82** Relieving after administration of drug

**Y83** Not relieving after administration of drug

**Y84** Relieving after warming

**X9** Edema

**Y91** Location of edema:

**Y911** Edema in Lower limbs

**Y912** Edema in head and face

**Y913** Edema in eyelid
**Y914** Edema from the lower limbs to all the body
**Y915** Edema from the face to all the body
**Y92** Character of edema:
**Y921** Rebounding after relief of pressure
**Y922** Pits under pressure
**X10** Hypodynamia
**X11** Dysphoria
**X12** Paroxysmal nocturnal dyspnea
**X13** Amnesia

2. **Common Inquiry**

**I Inquiry of cold and fever**:
**X14** Aversion to wind/Aversion to cold
**X15** Fear of cold
**X16** Cold limbs
**X17** Fevers
**Y171** Elevated body temperature
**Y172** Autopsyche fever without elevated body temperature
**X18** The tidal fever
**X19** Heat sensation in both palms and soles

**II Inquiry of perspiration:**
**X20** Spontaneous sweating
**X21** Night sweat

**III Inquiry of head, thorax and abdomen:**
**X22** Dizziness and Blurred vision
**X23** Tinnitus
**X24** Orolingual sore
**X25** Gingival swelling and pain
**X26** Gingival hemorrhage
**X27** Sore-throat
**X28** Cough
**X29** Cough with sputum
**Y291** Color of sputum:
**Y2911** Whitish sputum
**Y2912** Yellowish sputum
**Y2913** Dark grayish sputum
**Y2914** Blood in sputum
**Y2915** Ferruginous sputum
**Y2916** Pinkish sputum
**Y2917** Bloody purulent sputum
**Y2918** Cough with hemoptysis
**Y292** Character of sputum:
**Y2921** Thin sputum

**Y2922** Thick sputum
**Y2923** Frothy sputum
**Y293** Amount of expectoration:
**Y2931** Abundance sputum
**Y2932** Moderate sputum
**Y2933** Scanty sputum
**Y294** Difficulty or easy level of coughing with sputum:
**Y2941** Easy level of coughing with sputum
**Y2942** Difficulty level of coughing with sputum
**Y295** Taste of sputum:
**Y2951** Stinking smell of sputum
**Y2952** Sweet taste of sputum
**Y2953** Salty taste of sputum
**X30** Hiccups
**X31** Acid vomiting
**X32** Gastric stuffiness
**X33** Stomachaches
**Y331** Preference for warm and pressure
**Y332** Unpressable
**X34** Gastric upset
**X35** Heart-burn
**X36** Nausea and vomiting
**X37** Preference for sighing
**X38** Pain of hypochondrium
**X39** Abdominal fullness, distention and pain
**X40** Soreness and weakness of waist and knees
**X41** Numbness of hands and feet
**X42** Aching pain of whole body
**X43** Heavy sensation of body

## Ⅳ Inquiry of diet and taste

**X44** Thirsty and dry pharynx
**X45** Absence of thirst and no desire for water drink
**X46** Thirst with desire for water drink
**X47** Thirst with preference for cold water
**X48** Thirst with preference for hot water
**X49** Poor appetite and less amount of food
**X50** Good appetite but fast hunger
**X51** Hunger but no desire for food
**X52** Preference for food:
**Y521** Preference for sweet food
**Y522** Preference for salty food
**Y523** Preference for oily and sweet food
**Y524** Preference for hot and spicy food
**X53** Bitter taste
**X54** Sticky or greasy taste with a thick and greasy coating

**X55** Tastelessness with reduced appetite

## Ⅴ Inquiry of urination and defecation

**X56** Stool:__times/a day or__day/a time
**X57** Loose stool
**X58** Diarrhea
**X59** Stool with undigested food
**X60** Morning diarrhea
**X61** Irregular dry and loose stool
**X62** Constipation
**X63** Dry feces
**X64** Unsmooth defecation
**X65** Clear and profuse urine
**X66** Yellow and scanty urine
**X67** The frequent and urgent urination
**X68** Decreased urine
**X69** Unsmooth and painful urination
**X70** Urine with burning sensation
**X71** Dripping of urine
**X72** Frequent and increased urination at night

## Ⅵ Inquiry of sleep

**X73** Insommia
- **Y731** Condition of difficult in falling asleep
- **Y732** Condition of easy to be woken up after sleep
- **Y733** Condition of unable to fall asleep after woken up
- **Y734** Pernoctation
- **Y735** Dreamful sleep
- **Y736** Early awakening
- **Y737** Weakness after awakening

**X74** Drowsiness

## Ⅶ Inquiry of emotions

**X75** Impetuosity and susceptibility to rage
**X76** Susceptibility to fright
**X77** Susceptibility to anxiety and preoccuption
**X78** Susceptibility to sorrow

## Ⅷ Inquiry of women

**X79** Menopause
**X80** Menstruale cycle**:**
- **Y801** Advanced menorrhea
- **Y802** Delayed menorrhea
- **Y803** Irregular menstruation

**X81** Abnormal menstruation:
- **Y811** Profuse menstruation

**Y812** Scanty menstruation
**Y813** Amenorrhea
**Y814** Incessant dripping of menstrural blood
**Y815** Metrorrhagia

**X82** Menstrural color:
**Y821** Light reddish menstruation
**Y822** Dark reddish menstruation

**X83** The texture of menstruation:
**Y831** Thin menstruation
**Y832** Thick menstruation
**Y833** Menstruation with blood clot

**X84** Symptoms during menstruation:
**Y841** Distending pain of breast
**Y842** pain in lower abdomen
**Y843** Distending pain in lower abdomen
**Y844** Stabbing pain in lower abdomen
**Y845** Cold pain in lower abdomen
**Y846** Preference for warm and pressure

**X85** Leukorrhea:
**Y851** Whitish vaginal discharge
**Y852** Yellowish vaginal discharge
**Y853** Thin leukorrhea
**Y854** Thick leukorrhea

3. **History of past illness**

Disease history ___________________________________

Operation history (YES _________________NO)

Allergic history (YES_________________NO)
